# Nutritional Guideline for the Management of Mexican Patients with CKD and Hyperphosphatemia

**DOI:** 10.3390/nu12113289

**Published:** 2020-10-27

**Authors:** Frida Palafox-Serdán, Olinto A. Luna-Montiel, Sebastián E. Pablo-Franco, Daniela L. Guillen-Tejada, Sandra D. Carreño-Vázquez, Taísa S. Silva Pereira, Laura M. Islas Romero, Karen Villaseñor López, Ana E. Ortega-Régules, Aura M. Jiménez-Garduño

**Affiliations:** Health Sciences Department, Universidad de las Américas Puebla, UDLAP, Ex Hacienda Sta. Catarina Mártir S/N. Puebla, C.P. San Andrés Cholula 72810, Mexico; frida.palafoxsn@udlap.mx (F.P.-S.); olinto.lunaml@udlap.mx (O.A.L.-M.); sebastian.pablofo@udlap.mx (S.E.P.-F.); daniela.guillenta@udlap.mx (D.L.G.-T.); sandra.carrenovz@udlap.mx (S.D.C.-V.); taisa.silva@udlap.mx (T.S.S.P.); marissa.islas@udlap.mx (L.M.I.R.); karen.villasenor@udlap.mx (K.V.L.); ana.ortega@udlap.mx (A.E.O.-R.)

**Keywords:** Mexican food chart, hyperphosphatemia, chronic kidney disease, nutritional management

## Abstract

Chronic kidney disease (CKD) represents a serious concern for the Mexican population since the main predisposing diseases (diabetes, hypertension, etc.) have a high prevalence in the country. The development of frequent comorbidities during CKD such as anemia, metabolic disorders, and hyperphosphatemia increases the costs, symptoms, and death risks of the patients. Hyperphosphatemia is likely the only CKD comorbidity in which pharmaceutical options are restricted to phosphate binders and where nutritional management seems to play an important role for the improvement of biochemical and clinical parameters. Nutritional interventions aiming to control serum phosphate levels need to be based on food tables, which should be specifically elaborated for the cultural context of each population. Until now, there are no available food charts compiling a high amount of Mexican foods and describing phosphorus content as well as the phosphate to protein ratio for nutritional management of hyperphosphatemia in CKD. In this work, we elaborate a highly complete food chart as a reference for Mexican clinicians and include charts of additives and drug phosphate contents to consider extra sources of inorganic phosphate intake. We aim to provide an easy guideline to contribute to the implementation of more nutritional interventions focusing on this population in the country.

## 1. Introduction

In Mexico, chronic non-communicable diseases represent about 70% of all deaths [1] and one of the most urgent priorities for public health policies is chronic kidney disease (CKD). Since there is no centralized national registry of cases of CKD in Mexico, there are no precise reports of cases to date. However, it is well known that diabetes mellitus (DM) is the leading cause of CKD in both developing and developed countries [2]. The National Health and Nutrition Survey [3] reports that, in Mexico, DM was present in 10.3% (8.6 million) of the total population of the country aged 20 and over (82.7 million), representing an 1.1% increase in the last six years and many of those cases are not even aware of their kidney deterioration [1]. In addition, we need to consider other causes of CKD, such as systemic arterial hypertension (15.2 million adults in Mexico), autoimmune diseases, drug-related illnesses, etc., which also show an increasing-rate behavior. CKD is not included as such in the Universal Catalog of Health Services Causes of the public “Population Insurance” (Seguro Popular) in Mexico, leaving a significant percentage of the unprotected population left out of social security for CKD diagnosis and management. Around 124,000 patients with CKD due to diabetes in Mexico require renal replacement therapies (RRT) such as peritoneal dialysis, hemodialysis, and/or kidney transplantation, which is not covered by insurance [1].

CKD is defined by a Glomerular Filtration Rate (GFR) of <60 mL/mln/1.73 m^2^ and the presence of kidney damage, regardless of the cause, during a period of three or more months [4]. Among the pathologies associated with CKD are anemia, metabolic acidosis, heart disorders, and serum ion imbalances, such as hyperkalemia and hyperphosphatemia. In patients with CKD, the phosphate output stops working efficiently, leading to phosphate retention and disruption of the phosphate balance and leading to hyperphosphatemia [5], which is defined as a serum phosphate concentration above 4.5 mg/dL [6].

Hyperphosphatemia is a serious condition due to the amount of risk factors associated with high serum phosphate, which include increased mortality and cardiovascular disease [7]. The main consequence of hyperphosphatemia is vascular calcification, which initiates when calcium and phosphate bind and create hydroxyapatite molecules that deposit in arteries, myocardium, and cardiac valves. Other consequences of hyperphosphatemia are high levels of fibroblast growth factor 23 (FGF23), which is a hormone involved in phosphate and vitamin D regulation [8] and disruption of bone metabolism, leading to secondary hyperparathyroidism, renal osteodystrophy, and metabolic bone disease (CKD-MBD) [9]. Maintaining a strict control of phosphate levels in patients with CKD is imperative in order to avoid deterioration and associated risks. A 2017 publication by *Nature Reviews Nephrology* suggests dietary and pharmacological approaches for hyperphosphatemia treatment in patients suffering from CKD [8]. The first approach should be dietary, focusing on food selection, reducing the dietary phosphate intake, and considering the phosphate to protein ratio (PPR) of each food. It has been shown by many authors that a close follow-up during nutritional management is, by far, the safest and cheapest therapy able to reduce serum phosphate levels. However, educational aspects seem to play a key role to achieve good results. It is important to emphasize that Mexico is second place in adult obesity in the OECD countries (OECD reports 2017), which reflects the poor quality of nutritional habits of the population. This is the main concern in Mexico since implementing dietary changes for hyperphosphatemia treatment seems to be a very difficult task to achieve, above all, due to economic reasons. Anyhow, we believe that greater multidisciplinary efforts need to be done to improve treatment outcomes of patients with CKD and hyperphosphatemia. Above all, nutritional guidelines focusing on this specific population of CKD patients need to be available for local health care professionals in order to adapt management strategies in better ways.

Until now, we have found only three examples of indexed publications in Hispano-American countries aiming to condense and stratify the nutritional information based on food phosphate content. They represent local adapted food tables and guidelines for their specific populations and serve as a valuable tool for medical, nursing, and nutritional management in their respective countries [10,11,12].

According to our knowledge, there are only two publications aiming to approach the nutritional care of Mexican patients with hyperphosphatemia and CKD. The first one by Osuna et al. [13] is a review paper explaining the physiopathology of the disease, basic diet recommendations, most used phosphate binders, and cooking procedures to lower the phosphate content of foods. The second paper by Puchulu et al. [14] contains an animal source food table describing the PPR of most consumed foods in Mexico. Unfortunately, until now, Mexico’s national clinical practice guidelines for CKD management do not offer a specific section for high phosphate levels. Therefore, the present work aims to add efforts to the previously mentioned publications and to expand the food charts to all food groups, since Mexicans include many fruits, vegetables and cereals to prepare their dishes, and also to include equivalents to the chart. We present here a complete work of reference, which compiles the most consumed food groups with equivalents in Mexican society describing their PPR, information about phosphate based additives, and a summary of phosphate content in medications commonly used in CKD. Until low-cost and efficient pharmaceutical options appear in the market for hyperphosphatemia treatment, nutritional management is still our best option to control these patients and avoid further complications. To achieve better results, multidisciplinary working groups need to address the patients’ educational transition with close follow-ups. The present work wishes to contribute to the clinical practice moving toward that goal.

## 2. Materials and Methods

The nutritional information of each food presented was compiled through the following bibliographic sources: Pérez Lizaur AB, Palacios González B. Mexican System of Equivalent Foods for Kidney Patients. 1st ed. Mexico DF. Nutrition and health promotion. AC; 2009. Ledesma Solano JA, Chávez Villasana A, Pérez-Gil Romo F, Mendoza Martínez E, Calvo Carrillo C. Food composition Miriam Muñoz de Chávez. Nutritional value of the most consumed foods. 2nd ed. Mexico DF. McGraw-Hill; 2010. Todo Alimentos.org. Access via web: http://www.todoalimentos.org/.

For processed food, data from all available supermarkets in Puebla, Mexico were collected, as well as from the official web pages from all food brands included in the food tables. Products with missing data were not included. The following information was obtained from the photographs and online official pages: Name of the product, equivalent portion size and grams per portion, protein content (g), phosphorus content (mg) if described by the food company, potassium content (mg), sodium content (mg), and phosphorus food additives (if described by the food company).

PPR was calculated according to the data obtained for each food.

Regarding the data for drugs and additives, we focused on national databases complemented with previous published reports of phosphate content.

## 3. Results

Table 1 shows the nutrimental food content with equivalents, which focuses on CKD relevant data: protein, phosphorus, PPR, potassium, and sodium. We also added a column of “level of recommendation,” which is divided in three colors: red stands for “not recommended” for foods with a PPR above 16 mg/g, gray stands for “upon specialist criteria” for foods between 10 and 16 mg/g, and green stands for “recommended” for foods below 10 mg/g. The table, which includes 363 foods, is divided into the following groups: fruits, vegetables, type A cereals, type B cereals, legumes, animal source foods, oils and fats, others, and beverages. For most divisions, an additional table of “most consumed foods in Mexico” was included based on previous studies and on Mexican-supermarkets data collection. In the case of some processed foods, an additional column of phosphate-based additives was included. Most of the foods not included in this table had no known reported value of phosphorus content.

Until now, no strict threshold for PPR is accepted. Recommendations vary between 10 and 16 mg/g depending on each practitioner and author [10,15,16], which is why we categorized all foods with a PPR inside this range as possible to be consumed but only if recommended by a specialist. We believe that flexibility between 10 and 16 mg/g of PPR allows the nutritionist to widen the variety of foods without major risks of lowering protein and increasing phosphate intake. Since PPR aims to keep a protein-phosphate balance for the patient, it should be considered for protein-rich foods such as animal-derived products. In the case of fruits, vegetables, and cereals, we must focus on the net phosphate content to avoid misleading values of PPR. In case the food contains no phosphate and no protein, like the oils, then the PPR calculation is not possible. In cases where the food contains phosphorus but no protein, it is also not possible to calculate the ratio, but specialists should take care of phosphate food content in order to calculate daily consumption, which should stay below 700 mg/d [16], depending on the goal.

Table 2 shows the list of names of 25 phosphate-containing additives and how common they are when used in Mexico. It is very important that the names, as well as their code, are easily accessible for health professionals and patients in order to identify food sources of inorganic phosphates with high bioavailability.

Next, we present the last table (Table 3) showing the most common medications used in CKD patients in Mexico. Out of 24 drugs, only six drugs (25%) have known phosphate content. In Mexico, according to the NOM-072-SSA1-2012 (Mexican official policy), it is an optional requirement to describe the vehicle content of pharmaceuticals. Administrating medications with unknown phosphate content represent a potential source of inorganic phosphate that contributes to the phosphate load of the patients.

**Figure 1:**
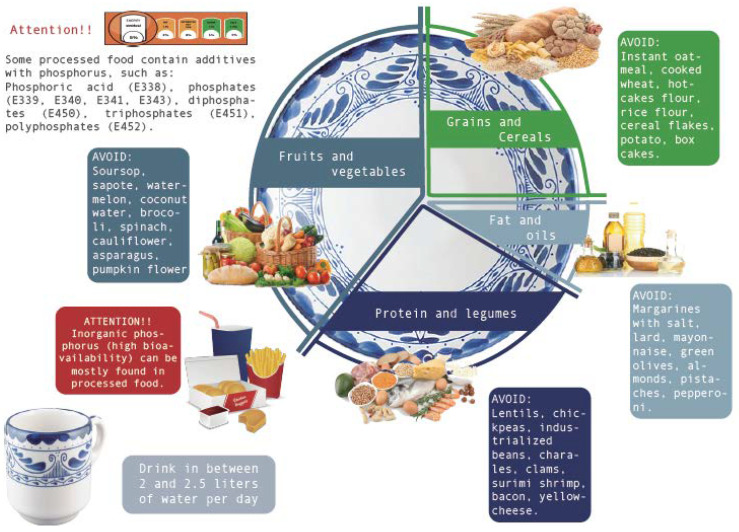
The Low Phosphate Plate.

Finally, we provide a graphical abstract entitled “The Low Phosphate Plate” (Figure 1). This abstract aims to provide visual guidance in order to remember which food types should be avoided, and emphasize the sources of inorganic phosphorus. The Low Phosphate Plate includes fruits and vegetables as the main food group, highlighting those that should be avoided. The other segments include cereals, animal-derived foods, and legumes. Here, the calculation of PPR becomes crucial in order to maintain a low phosphate intake without diminishing protein intake. The plate also includes oils and fats that are only recommended for consumption in small proportions.

## 4. Discussion

Phosphorus is contained in most nutrients, especially protein-rich foods, phytates (in plants), and food additives. A high protein content diet is strongly associated with a high phosphorus intake. Some previous recommendations in CKD suggest avoiding excess protein intake. However, low and very low protein diets may cause malnutrition, especially in patients with CKD, causing protein-energy wasting (PEW), and increasing risks for hospitalization, low quality life, and mortality [23].

The gastrointestinal absorption rate from plant-derived foods is between 10%–30%. In animal foods, it is up to 40%–60%, whereas phosphorus from inorganic sources found in medicines and additives has the highest absorption, up to 90%–100%, according to Noori et al. [16]. Vitamin D also affects the absorption because it can stimulate the expression of type IIb sodium-dependent phosphate transporters. On the other hand, nicotinamide functions as an inhibitor of intestinal phosphorus absorption [24].

A recently discovered way to reduce the phosphorus intake is to consider the PPR, which relates the phosphorus content per gram of protein. It has several advantages as a dietary management for patients with hyperphosphatemia in CKD, such as:

1. Its independent of the portion size or serving.
2. It focuses simultaneous attention on both proteins and phosphates, which are transcendental for the nutritional treatment of CDK.
3. The ratio allows you to choose from two similar options with different amounts of phosphorus but almost equal amounts of protein [16].

PPR is, therefore, a transcendental value when calculating the daily protein and phosphorus intake for these patients. The nutritionist should take special care of maintaining a proper protein intake including foods with low PPR so that they do not imply a high phosphate load, and also take care of low phosphate vegetables, fruits, and cereals for dishes’ preparation. For example, a typical Mexican dish called “Enchiladas” is prepared with chicken, tortilla, and a sauce (tomato and chili). For patients with hyperphosphatemia, the recommendation would be to use two wheat flour tortillas that have no phosphate content (instead of three corn tortillas typically used for the dish containing 282 mg of phosphorus), to use “poblano pepper” for the sauce, which contains only 9.5 mg of per portion, to use only one red tomato (12.4 mg of phosphorus), and, finally, to use 25 g of boiled breast chicken that has a PPR of 5.6 mg/g (without reusing the boiled water rich in phosphates). This version of the dish would provide 13.3 g of protein, 62 mg of phosphorus, and 527.4 mg of potassium for a final PPR of the dish of 4.6 mg/g, which is completely acceptable for the patient and preserves the cultural gastronomy.

Some studies suggest that plant-based diets can be effective to reduce the phosphorus concentration levels. However, increased phytate intake may cause deficiencies of some minerals such as iron, zinc, and calcium. Mexican gastronomy culture is not known for being well balanced, and the high overweight and obesity rates are a good reflection of it. Depending on the social status, nutrition can either include dishes predominantly from vegetable origin or with an excess of animal origin foods. A transition to achieve better nutrimental habits represents an enormous challenge, and specialized regional guidelines are the first steps toward the goal [25].

In many countries, including Mexico, food companies do not have an obligation to indicate the amount of phosphorus contained in their products nor the additives. Until new policies for nutritional labeling are established, all processed foods should be considered an important source of inorganic phosphate, unless otherwise specified.

Previous publications from Spain, Colombia, and Argentina [10,11,12] have shown the importance of compiling regional food charts for PPR calculations in order to improve nutritional management for patients with hyperphosphatemia. Those studies and the following to come from other countries represent a call from nutritional scientists to federal policies to take action toward better reports and regulations on phosphorus food content. Regarding the publications on the Mexican population, we have already mentioned the works by Osuna and Puchulu [13,14] that provide useful information for nutritional management of hyperphosphatemia in CKD. Compared to those publications, our work offers new content. We have expanded the chart to all food groups, and, although PPR is mainly useful for animal-derived foods, knowledge about phosphorus content in fruits, vegetables, and cereals is relevant for the calculations of dietary phosphorus intake below 700 mg/d. In addition, we offer a format with equivalents, which is the most used method for dietary management. Therefore, this version facilitates the use of the guide. We also provide a level of recommendation that offers a visual guidance of the best foods that can be included during the nutritional management. We provide the name and code of the most used additives with phosphates in the country so that specialists and patients can be aware of inorganic phosphate sources in the products of their choice. We also present a list of the most used medications in CKD patients, which show the lack of information regarding phosphate content in most of them, leaving the warning that the medications not reporting levels of phosphates should be taken as potential phosphorus sources. This is the first work to compile nutrimental and pharmaceutical information in such a complete way for the Mexican population. Finally, we provide an improved visual guide called the “low phosphate plate” (Figure 1), which depicts the dietary recommendations in a better way. Some previous works have shown “low phosphate pyramids” [11,14]. However, it has been nationally accepted that the pyramid designs do not clearly represent a nutritional recommendation since it depicts the least recommended foods at the top, while, for Mexicans, the top of a pyramid represents the most valuable place, which can lead to misunderstandings. Therefore, Mexico now uses the “plato del bien comer” (good eating plate) and, according to that same philosophy, we believe our “low phosphate plate” is a better representation of the dietary recommendations.

Nutritional education is an important aspect of clinical management to improve the patient’s lifestyle and, thereby, prevent the CKD-related complications. Martins et al. demonstrated that, after a four-month educational program based on the trans-theoretical model of behavioral change, serum phosphate levels decreased significantly, showing better results when combined with phosphate binders. The intervention consisted of lectures about nutrition presenting illustrative flip charts about food, phosphate binders, and the digestive system [26]. Other authors have also reported significant effects on serum phosphate levels after nutritional educational interventions [27].

There are, until now, no reported studies in Mexico analyzing the impact of nutritional interventions on biochemical and clinical parameters of patients with hyperphosphatemia and CKD. This may be partly due to the lack of bibliography and sources that summarize the current knowledge of food phosphate contents. Another important role of guidelines that systematize food phosphate content is to make evident the fact that this mineral is not regularly reported and represents a potential harm to all patients in the early stages of the disease. This represents increasing costs if hyperphosphatemia is developed. Regarding the benefits that this guide could provide to increase and improve nutritional interventions for serum phosphate control in CKD, we consider the following:

- To provide a complete food chart considering highly consumed products in Mexico of all food groups with an equivalent format and visual classification of the recommendation level of each food.
- To stress the necessity of new labeling policies in foods that could help complete the present food table and improve dietetic management of patients,
- To highlight the importance of phosphorus hidden in additives and medications often used in CKD, which contribute to the phosphorus load of the patients, and
- To use the image of our “Low Phosphate Plate” to spread the nutritional recommendations among our targeted patients throughout the country.

We are convinced that, with the use of this guide, the production of good quality pedagogic material for nutritional interventions will be easier and applicable either in individual private practice or in cohorts of patients in public clinics and hospitals. By using this guideline together with the previously cited Mexican works and proper pharmaceutical management, the control of phosphate serum can be enforced in the entire country with beneficial outcomes for the patients who already have hyperphosphatemia and as preventive measures for patients in the early stages of CKD.

## Figures and Tables

**Table 1:** Food table for Mexican population with phosphorus/protein ratio per portion.

**Food**	**Portion**	**Protein (g)**	**Phosphorus (mg)**	**Phosphorus/Protein Ratio**	**Level of Recommendation**	**Potassium (mg)**	**Sodium (mg)**	
Cactus Fruit	2 pieces (138 g)	1	4.3	4.3		302.5	0.1	
Grapefruit	1 piece (25 g)	0.9	5.3	5.8		225.7	0	
Cherry	20 pieces (88 g)	0.9	8.8	9.8		197.6	0	
Chopped pineapple	3/4 cup (124 g)	0.7	8.7	12.4		139.7	1.2	
Pineapple syrup	1 slice (50 g)	0.2	2.5	12.5		51.3	0.5	
Tangerine	2 pieces (128 g)	1	12.8	12.8		227.5	1.3	
Papaya	1 cup (140 g)	0.8	11.2	14		359.8	4.2	
Carabao mango	1 piece (145 g)	1.2	17.4	14.5		273.9	10.2	
Dry date	2 pieces (17 g)	0.3	4.9	16.4		108.2	0.5	
Fig	2 pieces (72 g)	0.6	10.1	16.8		167	0.7	
Mamey Sapote	1/3 piece (85 g)	1.4	23.8	17		191.5	-	
Orange	2 pieces (152 g)	1.4	25.8	18.5		276	1.5	
Grape	18 pieces (86 g)	0.6	11.2	18.6		158.5	1.7	
Apple porridge	3/4 bottle (85 g)	0.3	6	20		42.3	2.5	
Natural orange juice	1/2 cup (120 g)	0.8	18	22.5		240	1.2	
Ataulfo mango	1/2 piece (62 g)	0.3	6.8	22.7		96.3	0	
Apple	1 piece (106 g)	0.3	7.4	24.7		122.4	0	
Sapodilla sapota	1/2 piece (75 g)	0.3	7.5	25		144.3	9	
Lime	3 pieces (147 g)	1	26.5	26.5		203	2.9	
Strawberries	17 pieces (204 g)	1.4	38.8	27.7		312	2	
Blackberry	3/4 cup (108 g)	0.8	22.7	28.4		211.7	0	
Pear	1/2 piece (81 g)	0.3	8.9	29.7		101.2	0	
Guava	3 pieces (124 g)	1	31	31		352.7	3.7	
Black sapote	1/2 piece (93 g)	0.7	24.2	34.5		43.7	0	
Kiwi	1 1/2 piece (114 g)	1.2	45.6	38		376.4	5.7	
Cantaloupe	1/3 piece (179 g)	1.5	57.3	38.2		553.4	16.1	
Plantain	1/4 piece (49 g)	0.5	21.6	43.1		195.6	7.8	
Soursop	1 piece (238 g)	1	57.1	57.1		109	0	
Coconut water	1 1/2 cup (360 g)	1.1	244.8	222.5		529.2	90	
Chopped watermelon	1 cup (160 g)	1	452.8	452.8		186	3.2	
Creole mango	1 1/2 piece (162 g)	1.3	12.9	9.9		306.2	8.1	
Chopped pineapple	3/4 cup (124 g)	0.7	8.7	12.4		139.7	1.2	
Tangerine	2 pieces (128 g)	1	12.8	12.8		227.5	1.3	
Yellow peach	2 pieces (153 g)	1.4	18.3	13.1		301	0	
Bitter orange	2 pieces (152 g)	1.9	25.8	13.6		252	0	
Chopped papaya	1 cup (140 g)	0.8	11.2	14		359.8	4.2	
Grape	18 pieces (86 g)	0.6	11.2	18.6		158.5	1.7	
Apple	1 piece (106 g)	0.3	7.4	24.7		122.4	0	
Whole strawberries	17 pieces (204 g)	1.4	38.7	27.6		312.1	2	
Pear	1/2 piece (81 g)	0.3	8.9	29.7		101.2	0	
Guava	3 pieces (124 g)	1	31	31		352.7	3.7	
Grapefruit	1 piece (162 g)	0.9	34	37.8		225.7	0	
Valencian melon	1/3 piece (179 g)	1.5	57.3	38.2		553.4	16.1	
Chopped watermelon	1 cup (160 g)	1	452.8	452.8		186	3.2	
**Vegetables**								
**Food**	**Portion**	**Protein (g)**	**Phosphorus (mg)**	**Phosphorus/Protein Ratio**	**Level of Recommendation**	**Potassium (mg)**	**Sodium (mg)**	
Huauzontle	1/2 cup (40 g)	1.8	3.4	1.9		186.4	0	
Cooked chopped eggplant	3/4 cup (24 g)	0.6	3.6	6		184.1	0.2	
Poblano pepper	2/3 piece (43 g)	1.1	9.5	8.6		145.1	2.1	
Chopped jicama	1/2 cup (60 g)	0.4	3.6	9		90	3.6	
Raw beet	1/4 piece (39 g)	0.8	7.4	9.3		131	22.2	
Raw papaloquelite	2 cups (108 g)	1.9	18.3	9.6		659.9	-	
Cherry tomatoes	75 g	2.3	24	10.4		131.6	9.7	
Cooked nopal	1 cup (149 g)	2	23.8	11.9		291	29.8	
Tomato	120 g (113 g)	1	12.4	12.4		267.3	6.7	
Cooked chopped chard	1/2 cup (72 g)	1.9	23.7	12.5		654.5	128.8	
Raw watercress	1 piece (28 g)	0.8	11.2	14		112	3.6	
Cooked cauliflower	3/4 cup (94 g)	1.7	30	17.6		133.1	14.1	
Cooked spinach	1/2 cup (90 g)	2.7	50.4	18.7		419	63	
Raw asparagus	6 pieces (90 g)	2.3	46.8	20.3		144	1.8	
Cooked brussels sprout	3 pieces (63 g)	1.6	35.2	22		199.5	13.2	
Raw chopped coriander	1 3/4 cup (105 g)	2.2	57.7	26.2		547.1	21	
Cooked medium artichoke	1 piece (48 g)	1.4	36.9	26.4		137.3	38.4	
Cooked onion	1/4 cup (53 g)	0.7	18.5	26.4		87	1.6	
Cooked broccoli	1/2 cup (92 g)	2.7	75.4	27.9		268.9	51.5	
Lettuce	3 cups (135 g)	1.7	52.6	30.9		334.4	12.1	
Cooked pumpkin flower	1 cup (134 g)	1.4	45.5	32.5		142	8	
Raw celery	1 1/2 cup (135 g)	0.9	35.1	39		350.6	118.8	
Carrot juice	1/4 cup (59 g)	0.6	24.7	41.2		172.2	17.1	
Cooked Cushaw pumpkin	1/2 cup (110 g)	0.8	33	41.3		253	1.1	
Chopped cooked chayote	1/2 cup (80 g)	0.5	23.2	46.4		138.4	189.6	
Sliced cucumber	1 1/4 cup (130 g)	0.8	67.6	84.5		191.1	2.6	
Raw cheese	80 g (66 g)	3.1	291.7	94.1		400.8	13.2	
Chopped raw spinach	2 cups (120 g)	3.4	562.8	165.5		669.9	156	
Xoconostle	3 pieces (71 g)	0.1	29.1	291		155.7	-	
**Most Consumed Vegetables in the Mexican Population**								
**Food**	**Portion**	**Protein (g)**	**Phosphorus (mg)**	**Phosphorus/Protein Ratio**	**Level of Recommendation**	**Potassium (mg)**	**Sodium (mg)**	
Poblano pepper	2/3 piece (43 g)	1.1	9.5	8.6		145.1	2.1	
Cooked nopal	1 cup (149 g)	2	23.8	11.9		291	29.8	
Tomato	120 g (113 g)	1	12.4	12.4		267.3	6.7	
Cooked chopped chard	1/2 cup (72 g)	1.9	23.7	12.5		654.5	128.8	
Cooked cauliflower	3/4 cup (94 g)	1.7	30	17.6		188	14.1	
Cooked spinach	1/2 cup (90 g)	2.7	50.4	18.7		419	63	
Cooked broccoli	1/2 cup (92 g)	2.7	75.4	27.9		381	52.6	
Lettuce	3 cups (135 g)	1.7	52.6	30.9		334.4	12.1	
Cooked Castile squash	1/2 cup (110 g)	0.8	33	41.3		253	1.1	
Sliced white onion	1/2 cup (58 g)	0.5	23.2	46.4		87	2.3	
Chopped cooked chayote	1/2 cup (80 g)	0.5	23.2	46.4		138.4	189.6	
Sliced peeled cucumber	1 1/4 cup (130 g)	0.8	67.6	84.5		191.1	2.6	
**Type A Cereals**								
**Food**	**Portion**	**Protein (g)**	**Phosphorus (mg)**	**Phosphorus/Protein Ratio**	**Level of Recommendation**	**Potassium (mg)**	**Sodium (mg)**	
Powder Atole	7 teaspoons (18 g)	0	40	Not applicable		-	1	
Cornstarch	2 tablespoons (16 g)	0	35	Not applicable		-	1	
Tapioca	2 tablespoons (19 g)	0	1	Not applicable		-	0	
Wheat tortilla	1/2 piece (14 g)	1	0	0		-	105	
Cornflakes	1/3 cup (13 g)	1.1	5	4.5		-	163	
Hamburger bun	1/2 piece (26 g)	2.5	15	6		-	112	
Ground bread	8 teaspoons (16 g)	2.1	15	7.1		-	96	
Bagel	1/3 piece (24 g)	2.5	23	9.2		-	126	
Whole grain bagel	1/3 piece (24 g)	2.5	23	9.2		-	120	
Cooked wheat pasta	1/2 cup (60 g)	3.2	31	9.7		-	49	
Croutons	1/2 cup (15 g)	1.8	18	10		-	105	
Cooked spaghetti	1/3 cup (46 g)	2.5	25	10		-	1	
Bread sticks	3 pieces (18 g)	2.2	22	10		-	118	
Wheat flour	2 1/2 tablespoons (20 g)	2	21	10.5		-	1	
Low fat granola	3 tablespoons (18 g)	1.5	17.1	11.4		-	37	
Baguette	1/7 piece (27 g)	2.2	26	11.8		-	146	
Box bread	1 slice (27 g)	2.2	26	11.8		-	150	
Pretzels	3/4 cup (19 g)	1.7	21	12.4		-	325	
Roll	1/3 piece (20 g)	1.9	25	13.2		-	113	
Pambazo	1 piece (25 g)	2.4	32	13.3		-	142	
Animal cookie	6 pieces (15 g)	1.1	17	15.5		-	59	
Cooked sweet potato	1/4 cup (53 g)	0.9	14	15.6		-	7	
Cooked whole wheat spaghetti	1/3 cup (46 g)	2.5	41	16.4		-	1	
Cooked whole wheat pasta	1/3 cup (46 g)	2.5	41	16.4		-	1	
Pasta for soup	20 g	2.7	45	16.7		-	2	
Cooked rice	1/4 cup (47 g)	1.1	20	18.2		-	2	
Tortilla dough	45 g	1.6	35	21.9		-	1	
Cornmeal	2 1/2 tablespoons (18 g)	1.7	40	23.5		-	1	
Corn flour for tamales	2 1/2 tablespoons (18 g)	1.7	40	23.5		-	1	
Hot cake	3/4 piece (38 g)	2.5	59	23.6		-	165	
Whole wheat bread	1 slice (25 g)	2.4	57	23.8		-	146	
Whole wheat flour	2 1/2 tablespoons (19 g)	2.6	65	25		-	1	
Canned yellow corn	1/2 piece (82 g)	2.1	53	25.2		-	175	
Baked sweet potato	1/3 cup (70 g)	1.2	31	25.8		-	6	
Corn flour for atole	2 1/2 tablespoons (19 g)	1.5	41	27.3		-	1	
Oat bar	1/2 piece (14 g)	0.8	22	27.5		-	53	
Roasted amaranth	1/4 cup (16 g)	2.2	62	28.2		-	7	
Cooked yellow corn	1 1/2 piece (174 g)	2.2	68	30.9		-	2	
Hominy	1/3 cup (54 g)	1.8	56	31.1		-	9	
Rye	5 teaspoons (22 g)	2.6	81	31.2		-	1	
White corn shelled	1/2 cup (83 g)	2.6	85	32.7		-	4	
Cooked brown rice	1/3 cup (65 g)	1.5	54	36		-	1	
Cooked oatmeal	3/4 cup (164 g)	5.2	196	37.7		-	1	
Baked potato	1/2 piece (85 g)	1.5	63	42		-	4	
Instant oatmeal	2 tablespoons (28 g)	4.8	202	42.1		-	71	
Potato	3 pieces (105 g)	1.8	78	43.3		-	6	
Rice flour	2 tablespoons (20 g)	1.4	67	47.9		-	2	
Cooked wheat	1 1/2 tablespoons (21 g)	2.2	106	48.2		-	1	
Cereal flakes with dried fruit	1/3 cup (18 g)	1.3	83	63.8		-	110	
Corn tortilla	1 piece (30 g)	1.4	94	67.1		-	14	
Flour for hot cakes	2 tablespoons (18 g)	1.7	122	71.8		-	244	
Nixtamalized corn tortilla	1 piece (30 g)	1.3	94	72.3		-	14	
**Type B Cereals**								
**Food**	**Portion**	**Protein (g)**	**Phosphorus (mg)**	**Phosphorus/Protein Ratio**	**Level of Recommendation**	**Potassium (mg)**	**Sodium (mg)**	
Garlic bread	1 piece (28 g)	2	0	0		-	142	
Muffin	1 slice (45 g)	4	0	0		-	0	
Microwave Buttered Popcorn	2 1/2 cups (38 g)	3.5	0	0		-	384	
Cinnamon rolls with raisins	1/3 piece (29 g)	1.8	22	12.2		-	112	
Apple pie	1/3 slice (42 g)	0.8	10	12.5		-	111	
Seasoned croutons	3/4 cup (23 g)	2.4	32	13.3		-	278	
Cream cake	1/2 slice (23 g)	1.4	19	13.6		-	65	
Cheese pie	1/2 slice (28 g)	1.5	25	16.7		-	57	
Mac and cheese	1/4 cup (50 g)	4.2	81	19.3		-	272	
Commercial brownie	1/2 piece (28 g)	1.4	29	20.7		-	88	
Oatmeal cookie	1/3 piece (24 g)	1.5	33	22		-	92	
Mashed potatoes	1/2 cup (105 g)	2	48	24		-	333	
Chocolate-covered cookie	1 1/2 piece (21 g)	1.2	29	24.2		-	62	
Granola with almonds	3 tablespoons (21 g)	2.5	62	24.8		-	7	
Granola with raisins and dates	3 tablespoons (21 g)	2.5	62	24.8		-	5	
Frozen potato for frying	50 g	1.6	41	25.6		-	15	
Granola bar	3/4 piece (21 g)	2.2	59	26.8		-	62	
chips	6 pieces (18 g)	1.1	30	27.3		-	118	
corn chips	20 g	1.4	41	29.3		-	107	
Granola bar with raisins and walnuts	3/4 piece (21 g)	1.7	51	30		-	54	
Nachos	3 pieces (21 g)	1.7	52	30.6		-	151	
Carrot cake	1/2 slice (25 g)	1.3	44	33.8		-	89	
Chocolate-covered granola bar	3/4 piece (21 g)	1.2	42	35		-	42	
Glazed donut	1/3 piece (22 g)	1	35	35		-	74	
Chocolate cake	3/4 slice (38 g)	2.1	76	36.2		-	213	
Donut	1/3 piece (21 g)	1	58	58		-	117	
Sugared donut	1/3 piece (21 g)	1	58	58		-	117	
Lemon pie with meringue	1/3 slice (38 g)	0.6	40	66.7		-	55	
Bisquet	1/2 piece (33 g)	2	140	70		-	342	
**Most Consumed Cereals in The Mexican Population**								
**Food**	**Portion (grams)**	**Protein (g)**	**Phosphorus (mg)**	**Phosphorus/Protein Ratio**	**Level of Recommendation**	**Potassium (mg)**	**Sodium (mg)**	**Additives with Phosphorus**
Quaker Instant Oatmeal	1 bag (35 g)	2.8	130	2.9		-	382	-
Instant rice “knorr”	3/4 cup (125 g)	2.4		Not applicable			310	disodium iosinate and guanylate
Coconut bar “gamesa”	30 g	2	-	Not applicable		-	366	E340
Bar “All-bran”	37 g	3	-	Not applicable		-	188	E338
Chia multigrain bar “bimbo”	32 g	3	80	26.6		-	152	-
Cereal “Nesquik”	100 g	5.7	-	Not applicable		-	246	Dicalcium phosphate, trisodium phosphate
Cereal “Corn-Flakes”	100 g	7.5	102	13.6		168	729	Tricalcium phosphate, trisodium phosphate
**Legumes**								
**Food**	**Portion**	**Protein (g)**	**Phosphorus (mg)**	**Phosphorus/Protein Ratio**	**Level of Recommendation**	**Potassium (mg)**	**Sodium (mg)**	
Pea sprouts	1/2 cup (103 g)	7.2	25	3.5		275	3	
Bean sprouts	1 cup (190 g)	9.1	72	7.9		369	13	
Cooked beans	1/2 cup (90 g)	8.7	101	11.6		502	5	
Cooked dry pea or alverjón	1/2 cup (98 g)	8.2	97	11.8		355	2	
Lamapa black bean “green valley” (bagged bean)	48 g	10	125	12.5		-	160	
Stewed canned beans	1/3 cup (86 g)	6.3	79	12.5		393	300	
Soya flour	4 tablespoons (25 g)	9.3	117	12.6		500	3	
Cooked sprouted beans	1 cup (124 g)	8.8	128	14.5		393	17	
Cooked soy	1/3 cup (57 g)	9.4	139	14.8		292	1	
Average cooked beans	1/2 cup (86 g)	7.6	120	15.8		305	1	
Soybean sprouts	1 cup (94 g)	8	127	15.9		334	9	
Cooked beans	1/2 cup (85 g)	6.5	106	16.3		281	4	
Canned whole beans	1/2 cup (128 g)	6.7	115	17.2		303	379	
Canned chickpeas	1/2 cup (120 g)	6	108	18		207	359	
Cooked chickpeas	1/2 cup (82 g)	7.3	138	18.9		239	6	
Ibes or cooked lime beans	1/2 cup (85 g)	5.8	111	19.1		485	14	
Pinto beans “valle verde” (bean bag)	49 g	10	192	19.2		-	3	
Lentil sprouts	1 cup (77 g)	6.9	133	19.3		248	8	
Cooked lentils	1/2 cup (99 g)	9	178	19.8		366	2	
Black beans “green valley” (bagged bean)	47 g	10	238	23.8		-	161	
**Most Consumed Legumes in the Mexican Population**								
**Food**	**Portion**	**Protein (g)**	**Phosphorus (mg)**	**Phosphorus/Protein Ratio**	**Level of Recommendation**	**Potassium (mg)**	**Sodium (mg)**	
Chickpeas	1/2 cup (82 g)	7.3	138	18.9		239	6	
Cooked lentils	1/2 cup (99 g)	9	178	19.8		366	2	
Bean “Green valley”	1/4 cup (45 g)	9	199.2	22.1		-	8	
**Animal Source Foods**								
**Food**	**Portion**	**Protein (g)**	**Phosphorus (mg)**	**Phosphorus/Protein Ratio**	**Level of Recommendation**	**Potassium (mg)**	**Sodium (mg)**	
Egg whites	2 pieces (66 g)	7.2	10	1.4		95	110	
Dried beef	11 g	7.1	19	2.7		48.8	382	
Beef legs	120 g	6.7	30	4.5		-	309	
Smoked white fish	32 g	7.5	42	5.6		-	326	
Roast chicken breast	25 g	7.2	40	5.6		-	16	
Cooked chicken breast	25 g	7.2	40	5.6		-	16	
Cooked chicken	25 g	7.2	40	5.6		-	16	
Cooked groupers	30 g	7.5	43	5.7		-	16	
Corned beef	25 g	6	37	6.2		74.3	275	
Spicy beef Cecina	25 g	6	37	6.2		-	275	
Cooked chicken gizzards	25 g	7.6	47	6.2		-	14	
Tuna in water	1/5 cup 31 g)	7.9	50	6.3		-	104	
Light tuna	1/5 cup (31 g)	7.9	50	6.3		-	104	
Cooked shrimp	5 pieces (34 g)	7.1	47	6.6		41.5	76	
Guinea fowl with fur	30 g	5.8	38	6.6		-	17	
Chicken tuna without skin	1 piece (29 g)	6.4	45	7		-	24	
Leg of lamb	30 g	6.4	46	7.2		-	15	
Kid (lamb)	25 g	6.8	50	7.4		-	22	
Cooked pork heart	25 g	5.9	45	7.6		-	9	
Cooked red snapper	28 g	7.4	56	7.6		-	16	
Cooked red snapper	28 g	7.4	56	7.6		-	16	
Shredded cooked crab	1/3 cup (47 g)	8.1	65	8		124.6	19	
Chicken Milanese	30 g	7	56	8		-	20	
Ground chicken	32 g	7.4	60	8.1		-	22	
Raw skinless chicken fajita	1 1/3 piece (33 g)	6.8	55	8.1		-	29	
Crab pulp	1/3 cup (46 g)	8	65	8.1		-	19	
Guinea fowl without skin	55 g	7.2	59	8.2		-	24	
Raw skinless chicken leg	1/4 piece (33 g)	6.6	55	8.3		-	28	
Fresh anchovies	30 g	6.1	52	8.5		-	31	
Raw chicken thighs without skin	2/5 piece (36 g)	7.1	60	8.5		-	31	
Raw skinless chicken thighs	1/2 piece (34 g)	6.8	58	8.5		-	30	
Aguayón	30 g	6.3	54	8.6		107.4	19	
Cooked beef hearts	20 g	5.8	50	8.6		-	12	
Steak	30 g	7.2	63	8.8		-	18	
Beef steak	30 g	7.2	63	8.8		-	18	
Mignon steak	1/4 piece (30 g)	7.2	63	8.8		-	18	
Tampiqueña steak	1/4 piece (30 g)	7.2	63	8.8		-	18	
Steak medallions	1/4 piece (30 g)	7.2	63	8.8		-	18	
Breaded beef	30 g	7.2	63	8.8		-	18	
Fillet tips	30 g	7.2	63	8.8		-	18	
Beef tips	30 g (24 g)	7.2	63	8.8		-	18	
Tampiqueña	1/4 piece (30 g)	7.2	63	8.8		-	18	
Smoked salmon	35 g	6.4	57	8.9		-	274	
Cooked beef hearts	25 g	7.1	64	9		-	15	
Cooked lobsters	35 g	7.2	65	9		-	133	
Shank	35 g	6.8	62	9.1		105	20	
Dry shrimp	10 g	7.5	70	9.3		-	221	
Salty dried shrimp	10 g	7.5	70	9.3		-	221	
Cooked octopus	25 g	7.5	70	9.3		-	115	
Cooked pork kidneys	28 g	7.1	67	9.4		-	22	
Raw anchovy	45 g	6.4	63	9.8		-	37	
Fish steak	40 g	7.5	74	9.9		-	32	
Filleted fish	40 g	7.5	74	9.9		-	32	
Cooked snook	30 g	7.3	72	9.9		-	26	
Ground turkey	33 g	7.4	75	10.1		-	473	
Turkey breasts	1 1/2 slice (32 g)	7.1	72	10.1		-	452	
Cooked crab	1/3 cup (39 g)	8	81	10.1		-	110	
Cooked perch	30 g	7.4	77	10.4		-	24	
Beef balls	35 g	7.3	77	10.5		-	22	
Cooked chicken giblets	25 g	6.8	72	10.6		-	17	
Baked tuna	20 g	6	65	10.8		-	10	
Cottage cheese	3 tablespoons (48 g)	6.6	72	10.9		-	195	
Low fat cottage cheese	1/4 cup (57 g)	7	76	10.9		-	230	
Fresh tuna	30 g	7	76	10.9		-	12	
Fresh abalone	40 g	6.8	76	11.2		-	120	
Cooked beef kidneys	25 g	6.8	76	11.2		-	24	
Fresh fish cod	45 g	8	92	11.5		-	24	
Beef belly	45 g	6.2	73	11.8		-	20	
Cooked shelled mussels	25 g	5.9	71	12		-	92	
Cooked turkey hearts	30 g	6.4	78	12.2		-	27	
Fresh clam without shells	4 pieces (58 g)	7.4	98	13.2		136.3	33	
Cooked swordfish	28 g	7.1	94	13.2		-	32	
Roast beef	2 1/2 slices (33 g)	6.6	90	13.6		-	329	
Smoked ham	2 thin slices (42 g)	7	98	14		-	510	
Squid rings	1/4 cup (38 g)	5.9	83	14.1		-	16	
Cooked veal kidneys	25 g	6.6	93	14.1		-	28	
Cooked ham	2 thin slices (42 g)	7	100	14.3		-	566	
Clean fresh squid	45 g	7	100	14.3		-	20	
Shrimp	110 g	7.2	106	14.7		402.6	181	
Dried codfish	13 g	7.4	111	15		-	822	
Low sodium ham	2 thin slices (36 g)	5.9	91	15.4		-	301	
Smoked turkey breasts	1 1/2 slice (34 g)	6.7	107	16		-	403	
Low-fat smoked turkey breasts	2 1/3 slice (49 g)	7.3	119	16.3		-	535	
Smoked pork chops	1/2 piece (38 g)	6.1	105	17.2		-	457	
Canadian Bacon	3 slices (45 g)	8	139	17.4		-	605	
Surimi	2/3 bar (40 g)	6	112	18.7		-	57	
Cambarellus	50 g	7.4	148	20		-	0	
Surimi shrimp	40 g	5	113	22.6		-	282	
Imitation shrimp surimi	40 g	4.9	113	23.1		-	282	
Dry charales	15 g	8.2	314	38.3		-	294	
Clam juice	7 cups (1680 g)	7	1918	274		-	3612	
**Most Consumed Animal Source Foods In The Mexican Population**								
**Food**	**Portion**	**Protein (g)**	**Phosphorus (mg)**	**Phosphorus/Protein Ratio**	**Level of Recommendation**	**Potassium (mg)**	**Sodium (mg)**	
Dried beef (machaca)	11 g	7.1	19	2.7		48.8	382	
Chicken breast	25 g	7.2	40	5.6		-	16	
Shrimp	5 pieces (34 g)	7.1	47	6.6		41.5	76	
Pork meat	25 g	5.9	45	7.6		-	9	
Raw skinless chicken legs	1/4 piece (33 g)	6.6	55	8.3		-	28	
Beef	30 g	7.2	63	8.8		-	18	
Fresh fish	40 g	7.5	74	9.9		-	32	
Chicken feet	25 g	6.8	72	10.6		-	17	
Fresh cod	45 g	8	92	11.5		-	24	
**Oils And Fats**								
**Food**	**Portion (g)**	**Protein (g)**	**Phosphorus (mg)**	**Phosphorus/Protein Ratio**	**Level of Recommendation**	**Potassium (mg)**	**Sodium (mg)**	
Oil	1 teaspoon (5 g)	0	0	Not applicable		-	0	
Canola oil	1 teaspoon (5 g)	0	0	Not applicable		-	0	
Sunflower oil	1 teaspoon (5 g)	0	0	Not applicable		-	0	
Corn oil	1 teaspoon (5 g)	0	0	Not applicable		-	0	
Olive oil	1 teaspoon (5 g)	0	0	Not applicable		-	0	
Pepperoni	4 slices (15 g)	2.8	22.4	8		52.1	274	
Skin avocado	1 1/2 piece (36 g)	0.5	7	14		308	1	
Roasted peanut	13 pieces (12 g)	2.8	46	16.4		79	96	
Almond	10 pieces (12 g)	2.7	62.4	23.1		92.8	1	
Pistachio	18 pieces (13 g)	2.6	65.4	25.2		126.4	86	
Pitted green olives	8 pieces (24 g)	0.3	17	56.7		55	236	
Margarine with salt	1 teaspoon (6 g)	0.1	22.9	229		42	61	
Butter with salt	1 1/2 teaspoon (6 g)	0.1	23	230		26	54	
Unsalted butter	1 1/2 teaspoon (4 g)	0.1	23	230		10	1	
Mayonnaise	1 teaspoon (5 g)	0.1	28	280		-	28	
**Other (Snacks, Aperitives)**								
**Food**	**Portion (g)**	**Protein (g)**	**Phosphorus (mg)**	**Phosphorus/Protein Ratio**	**Level of Recommendation**	**Potassium (mg)**	**Sodium (mg)**	
Candy, hard or jelly beans	10 pieces (80 g)	1.2	1.0	0.8				
Popsicle, fruit and juice bars	30 g	1.2	5.0	4.2		8.0	4.0	
Cookie: chocolate chip or sugar (medium size)	1 piece (15 g)	2.0	15.0	7.5		30	60	
Candy, caramels	1 piece (10 g)	0.0	10.0	10				
Gelatin/Jell-O^®^	1/2 cup (113 g)	2.0	30.0	15.0		-	43.5	
Brownie (2″ square)	1 slice (50 g)	2.8	55	19.6		61.0	175.0	
Candy bar, white chocolate (turion)	28 g	2.0	50.0	25.0		-	35.0	
Whipped topping, frozen, fat-free	1/2 cup (113 g)	1.1	30.0	27.3		38.0	27.0	
Nutella (chocolate-flavored hazelnut spread)	2 tablespoons (37 g)	2.0	55.0	27.5		-	15.0	
Sherbet	1/2 cup (113 g)	1.0	30.0	30.0		92.5	44.5	
Popcorn, air or oil popped, regular or microwave	1 cup (70 g)	0.8	25.0	31.3		21.0	88.0	
Ice cream, soft serve, or frozen chocolate	1/2 cup (113 g)	2.5	100.0	40.0		-	50.1	
Ice cream, soft serve, or frozen yogurt	1/2 cup (113 g)	2.4	100.0	41.7		128.0	66.5	
Ice cream, soft serve, or frozen vanilla	1/2 cup (113 g)	2.3	100.0	43.5		-	52.8	
Candy bar, milk or dark chocolate	28 g	1.8	85	47.2		-	28.0	
Pudding, vanilla, ready-to-eat	1/2 cup (113 g)	0.9	45.0	50.0		128.0	53.0	
Pudding, chocolate, ready- to-eat	1/2 cup (113 g)	0.9	65.0	72.2		-	59.8	
Cocoa, dry powder	2 tablespoons (10 g)	1.0	80.0	80.0		0.0	1.0	
**Beverages**								
**Food**	**Portion (g)**	**Protein (g)**	**Phosphorus (mg)**	**Phosphorus/Protein Ratio**	**Level of Recommendation**	**Potassium (mg)**	**Sodium (mg)**	
Cola-type beverages	340 g	0.0	35.0	Not applicable		3.4	13.6	
Non-cola beverages, all types	340 g	0.0	0.0	Not applicable		-	93.0	
Beer, regular	340 g	40.8	60.0	1.5		85.0	17.0	
Tea, black or herbal	1 cup (226 g)	0.3	2.0	6.7		-	-	
Coffee, black, brewed	1 cup (226 g)	0.3	5.0	17.9		111.0	5.0	
Wine, red or white	1/2 cup (113 g)	0.2	25.0	125.0		100.6	9	

Level of recommendation: green = recommended, gray = upon specialist criteria, red = not recommended. The nutritional information of each food presented was compiled from refs. [17,18,19,20,21,22]. The nutritional information of processed foods was compiled through their official pages of the respective brands and from some tours to self-service stores such as Bodega Aurrera, Wal-Mart de México, and Tiendas Gran Bodega.

**Table 2:** Most frequent additives found in Mexican processed foods.

Code	Name	Use in Mexican Products
E340	Potassium orthophosphates	Very common
E340i	Monopotassium dihydrogen phosphate	
E340ii	Dipotassium mono-hydrogen phosphate	
E341	Calcium phosphates	
E341i	Monocalcium phosphate	
E450	Di-phosphates	
E450i	Di-sodium di-phosphate (di-sodium pyrophosphate)	
E101ii	Riboflavin-5-Sodium Phosphate	Uncommon
E338	Phosphoric acid	
E339	Sodium orthophosphates	
E339i	Monosodium di-hydrogen phosphate	
E339ii	Disodium mono-hydrogen phosphate	
E339iii	Tri-sodium phosphate	
E340iii	Tri-potassium phosphate	
E341ii	Calcium hydrogen phosphate	
E341iii	Tri-calcium phosphate	
E442	Ammonium phosphatides	
E450ii	Tri-sodium di-phosphate	
E450iii	Tetra-sodium pyrophosphate	
E451	Tri-phosphates	
E451i	Penta-sodium tri-phosphate	
E452	Polyphosphates	
E452i	Sodium polyphosphate	
E541	Sodium aluminium phosphate	
E1414	Acetylated di-starch phosphate	

**Table 3:** Most common medications used in chronic kidney disease (CKD) patients and phosphorus content description.

Medication	Class	Manufacturer Reporting P Content	Phosphorus Content (mg)
Lisinopril	Angiotensin-converting enzyme inhibitors	Merck	21.4/10 mg tablet
Enalapril			NA
Captopril			NA
Losartán	Angiotensin II receptor blockers		NA
Valsartán			NA
Propanol	β—blockers		NA
Bisoprolol			NA
Metoprolol			NA
Atenolol			NA
Rosuvastatin	Statins	AztraZeneca	1.8/10 mg tablet
Artrovastatin			NA
Fluvastatin			NA
Bezafibrate	Fibrate		NA
Ezetimibe	Cholesterols absorption inhibitors		NA
Canaglifozin	Sodium-glucose co-transporters 2		NA
Dapagliflozin			NA
Empaglifozin			NA
Metformin	Biguanides		NA
Glyburide	Sulfonylurea	Aurobindo	27.6/5 mg tablet
Repaglinide 1 mg	Meglitinides	Caraco	9.4/1 mg tablet
Sitagliptin 25 mg	DPP-4 inhibitors	Merck	7.3/25 mg tablet
Sitagliptin 50 mg		Merck	9.4/50 mg tablet
Furosemide	Loop diuretic		NA
Spironolactone	Potassium-sparing diuretics		NA
